# ATAD2 is a driver and a therapeutic target in ovarian cancer that functions by upregulating CENPE

**DOI:** 10.1038/s41419-023-05993-9

**Published:** 2023-07-21

**Authors:** Praveen Guruvaiah, Suresh Chava, Chiao-Wang Sun, Nirupama Singh, Courtney A. Penn, Romi Gupta

**Affiliations:** 1 Department of Biochemistry and Molecular Genetics, The University of Alabama at Birmingham, Birmingham, AL 35233 USA; 2 Department of Pathology, Division of Laboratory Medicine, University of Alabama at Birmingham, Birmingham, AL 35233 USA; 3 Division of Gynecologic Oncology, Department of Obstetrics and Gynecology, Vanderbilt University Medical Center, Nashville, TN 37232 USA; 4 O’Neal Comprehensive Cancer Center, The University of Alabama at Birmingham, Birmingham, AL 35233 USA

**Keywords:** Ovarian cancer, Cancer epigenetics

## Abstract

Ovarian cancer is a complex disease associated with multiple genetic and epigenetic alterations. The emergence of treatment resistance in most patients causes ovarian cancer to become incurable, and novel therapies remain necessary. We identified epigenetic regulator ATPase family AAA domain-containing 2 (ATAD2) is overexpressed in ovarian cancer and is associated with increased incidences of metastasis and recurrence. Genetic knockdown of *ATAD2* or its pharmacological inhibition via ATAD2 inhibitor BAY-850 suppressed ovarian cancer growth and metastasis in both in vitro and in vivo models. Transcriptome-wide mRNA expression profiling of ovarian cancer cells treated with BAY-850 revealed that ATAD2 inhibition predominantly alters the expression of centromere regulatory genes, particularly centromere protein E (*CENPE*). In ovarian cancer cells, changes in CENPE expression following ATAD2 inhibition resulted in cell-cycle arrest and apoptosis induction, which led to the suppression of ovarian cancer growth. Pharmacological CENPE inhibition phenotypically recapitulated the cellular changes induced by ATAD2 inhibition, and combined pharmacological inhibition of both ATAD2 and CENPE inhibited ovarian cancer cell growth more potently than inhibition of either alone. Thus, our study identified ATAD2 as regulators of ovarian cancer growth and metastasis that can be targeted either alone or in combination with CENPE inhibitors for effective ovarian cancer therapy.

## Introduction

Ovarian cancer is the leading cause of death among women with gynecological malignancies [1, 2]. Due to the lack of effective screening and non-specific early symptoms, ovarian cancer is often detected at advanced stages [3]. Treatment of primary advanced ovarian cancer typically involves both surgery and chemotherapy [4]. Although remission is achieved in most patients, disease recurrence is common, and recurrent ovarian cancer is often resistant to conventional therapies, including chemotherapy; angiogenesis inhibitors, such as bevacizumab; and poly (ADP-ribose) polymerase (PARP) inhibitors [5, 6]. Therefore, new approaches for ovarian cancer prevention, screening, detection, and treatment are needed to improve overall patient survival.

A characteristic common to cancer cells is the deregulation of genetic and epigenetic factors, which contribute to uncontrolled proliferation, even under unfavorable conditions [7, 8]. Epigenetic regulators play important roles in tumor growth, metastasis, and the response to cancer therapies, including the development of drug resistance [9], making them potential therapeutic targets. Currently, many inhibitors targeting epigenetic regulators are in clinical use, either as single-agent therapies or in combination with other anti-cancer agents [9].

Ovarian cancer cells undergo several epigenetic changes, including histone methylation and acetylation, leading to the acquisition of highly invasive, metastatic, and chemo-resistant properties [10, 11]. For instance, DNA hypermethylation in ovarian cancer cells results in the silencing of tumor suppressor genes, such as *BRCA1* and *PTEN*, whereas DNA hypomethylation activates oncogenes, including *HRAS*, *HMGA2*, *BCL2*, and *BCL3* [11]. Ovarian cancer relapse and chemo-resistance have been linked to epigenetic changes. For example, DNA methylation-induced silencing of MLH1 mismatch repair genes was associated with the relapse of a chemo-resistant ovarian tumor and silencing of the frizzled-related protein 5 (*SFRP5*) was associated with platinum resistance in ovarian cancer [11]. Based on these studies, epigenetic inhibitors targeting DNA methyltransferase and histone deacetylase are tested for treating recurrent chemo-resistant ovarian cancer, the treatment of which remains among the biggest challenges in ovarian cancer therapy [12, 13].

ATPase family AAA domain-containing protein 2 (ATAD2), a member of the ATPases (AAA^+^) family, is a highly conserved protein predominantly expressed in germ cells [14]. ATAD2 is an epigenetic regulator that functions as a co-factor for oncogenic transcription factors [15]. The bromodomain module of ATAD2 is essential for the association of ATAD2 with acetylated chromatin and is thought to be involved in ATAD2-mediated function [16]. Recent studies have demonstrated that ATAD2 is overexpressed in several cancer types [17–23] and plays a role in the regulation of key oncogenes, such as *c-Μyc* and *E2F1* [24], in genome regulation, cell proliferation, differentiation, and apoptosis [25, 26]. Thus, ATAD2 has been shown to be an important driver of tumor growth and progression. Based on these findings, new and potent small-molecule inhibitors targeting ATAD2 are currently being tested as therapeutic agents in various cancer types [27, 28]. However, in ovarian cancer, the role played by ATAD2 and whether targeting ATAD2 has therapeutic value is not well understood.

Here, we demonstrate that ATAD2 is overexpressed in ovarian cancer, and ATAD2 overexpression predicts metastatic disease progression and disease recurrence in patient-derived ovarian cancer samples. Furthermore, we show that ATAD2 inhibition suppresses tumor growth and metastasis in both in vitro and in vivo models of ovarian cancer. Finally, we demonstrate that the combined use of both ATAD2 and centromere protein E (CENPE) inhibitors results in a more potent suppressive effect on ovarian cancer growth than the use of either inhibitor alone. Collectively, our studies illuminate the role of ATAD2 as a facilitator of ovarian cancer growth and metastasis and indicate that ATAD2 inhibitors, used either alone or in combination with CENPE inhibitors, may represent therapeutic option for treating ovarian cancer patients.

## Results

### ATAD2 is overexpressed in patient-derived ovarian cancer samples, and ATAD2 overexpression is associated with poor prognosis

**Fig. 1:**
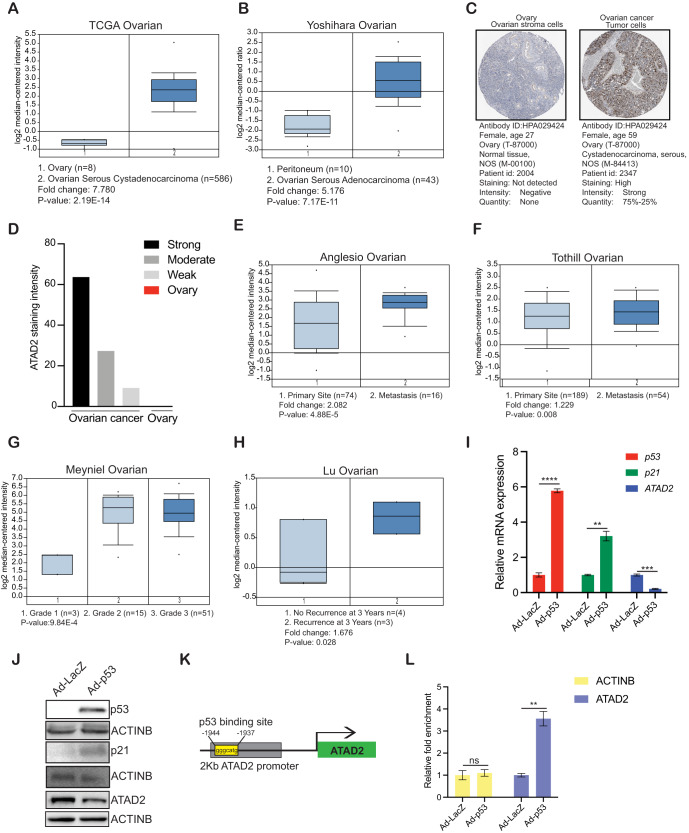
ATAD2 is overexpressed in patient-derived ovarian cancer samples. **A**, **B** ATPase family AAA domain-containing 2 (*ATAD2*) mRNA expression was analyzed in the indicated data sets from patients with ovarian cancer using Oncomine. *ATAD2* upregulation was observed in ovarian cancer samples relative to normal samples. **C**, **D** Representative images for the stained ovarian cancer patient samples and normal ovary using Human Protein Atlas (C) and ATAD2 protein expression is plotted in ovarian cancer patient samples and normal ovary using Human Protein Atlas (D). **E**, **F** *ATAD2* mRNA expression at primary sites and metastasis is plotted for the indicated datasets using Oncomine. **G** *ATAD2* mRNA expression at different disease grades is plotted for the indicated datasets using Oncomine. **H** *ATAD2* mRNA expression in patient with recurrence and no recurrence after 3 years is plotted for the indicated datasets using Oncomine. **I** Transcript levels for *ATAD2*, *p53*, and *p21* were evaluated by quantitative reverse-transcriptase-polymerase chain reaction (qRT-PCR) in SK-OV3 cells infected with adenovirus expressing p53 (Ad-p53) or control adenovirus expressing β-galactosidase (Ad-LacZ). **J** Protein levels for ATAD2, p53, and p21 were evaluated by immunoblotting in SK-OV3 cells infected with adenovirus expressing p53 (Ad-p53) or control adenovirus expressing β-galactosidase (Ad-LacZ). **K** Schematics showing p53 binding site on *ATAD2* promoter region. **L** SK-OV3 cells expressing Ad-p53 or Ad-LacZ were analyzed using CUT & RUN assay to evaluate the binding of p53 on *ATAD2* promoter. Data represent the mean ± standard error for three biological replicates. **p < 0.01, ***p < 0.001, ****p < 0.0001, ns: not significant.

ATAD2 can be targeted to achieve tumor inhibition and therapeutic benefits in some cancers [29–31]. However, in ovarian cancer, the contributions of ATAD2 to disease progression and the value of therapeutic ATAD2 targeting remain unknown. To understand the role of ATAD2 in ovarian cancer, we first analyzed *ATAD2* mRNA expression levels in ovarian cancer patient samples. Our analysis of several publicly available mRNA expression data sets [32–34] revealed that *ATAD2* is significantly overexpressed in patient-derived ovarian cancer samples (Fig. 1A, B) as compared to normal ovary tissue samples. Additionally, analysis of Human Protein Atlas data sets revealed that ATAD2 protein is also overexpressed in the majority of patient-derived ovarian cancer samples, in agreement with the mRNA expression data (Fig. 1C, D). Our analysis of other publicly available ovarian cancer data sets [35–38] revealed that *ATAD2* mRNA expression was higher in metastatic and high-grade ovarian cancer samples (Fig. 1E-G) and was associated with increased incidence of recurrence in patients (Fig. 1H). Collectively, these results demonstrate that ATAD2 is overexpressed in ovarian cancer samples at both the mRNA and protein levels and is associated with metastatic progression. Further, ATAD2 overexpression predicts poor patient prognosis.

We next interrogated the mechanism by which ATAD2 expression is upregulated in ovarian cancer. The tumor suppressor p53 is inactivated by either deletion or mutation in approximately 95% of all ovarian cancer cases [39]. Therefore, we investigated whether p53 is involved in the regulation of *ATAD2* expression. We infected SK-OV3 cells, which do not express endogenous p53 [40], with recombinant adenoviruses containing either the p53 gene (Ad-p53) or the β-galactosidase gene (Ad-LacZ, control). We observed that ectopic expression of p53 but not LacZ resulted in the downregulation of *ATAD2* mRNA levels and protein levels (Fig. 1I, J). To determine whether p53 directly regulates *ATAD2* transcription, we first analyzed the *ATAD2* promoter sequence using rVista2.0, which predicts potential DNA-binding sites for transcription factors. A DNA-binding site for p53 was identified in the *ATAD2* promoter (Fig. 1K). We then performed CUT & RUN assay to establish the association of p53 with the endogenous *ATAD2* promoter in SK-OV3 cells expressing either Ad-p53 or Ad-LacZ. Our results confirmed that p53 binds directly to the *ATAD2* promoter (Fig. 1L) and cause its transcriptional repression. These results demonstrate that the transcription factor p53 regulates ATAD2 expression in ovarian cancer cells.

### ATAD2 inhibition suppresses tumor growth and metastasis in cell culture models of ovarian cancer

**Fig. 2:**
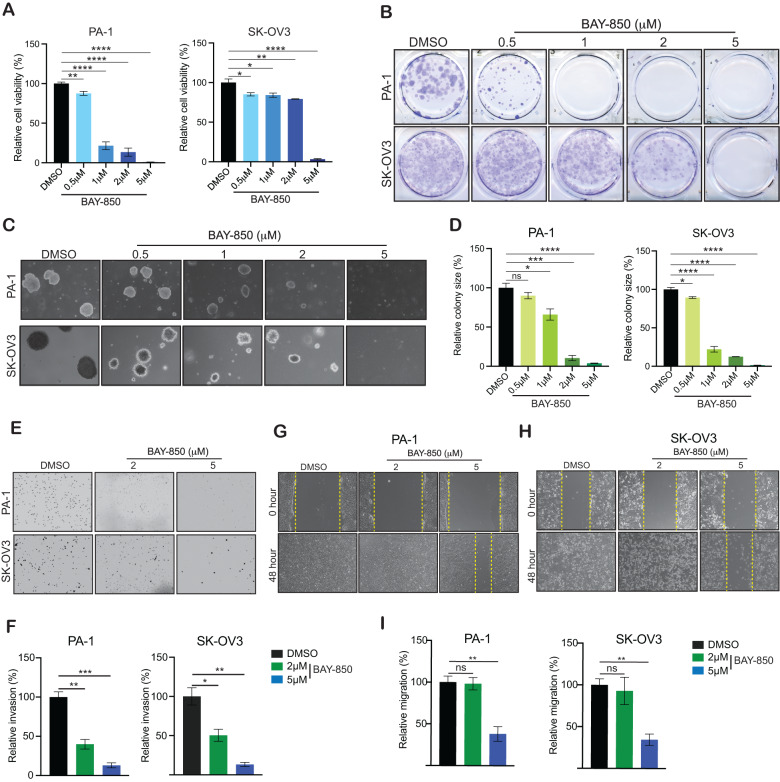
ATAD2 targeting inhibits ovarian cancer tumor growth. **A** The indicated ovarian cancer cell lines were treated with various concentrations of BAY-850 for 3 days, and survival was assessed by 3-(4,5-dimethylthiazol-2-yl)-2,5-diphenyltetrazolium bromide (MTT) assay. Cell survival is presented relative to the survival of DMSO-treated cells. **B** The indicated ovarian cancer cell lines were treated with various concentrations of BAY-850 for 2–4 weeks. Cell survival was measured using clonogenic assays. Representative images are shown. **C** The indicated ovarian cancer cell lines were treated with various concentrations of BAY-850 and analyzed for their abilities to grow in soft-agar assays. Representative images are shown; scale bar, 500 µm. **D** Relative colony sizes for the images shown in (**C**). **E** The indicated ovarian cancer cell lines were treated with various concentrations of BAY-850 and analyzed for invasive ability using Matrigel-based Boyden chamber assays. Representative images are shown; scale bar, 200 µm. **F** Percentage invasion for the images shown in (**E**). **G**, **H** Migration was analyzed in a wound-healing assay for ovarian cancer cell lines, PA-1 (**G**) and SK-OV3 (**H**), treated with various concentrations of BAY-850. Representative images are shown; scale bar, 200 µm. **I** Quantitation of the data presented in (**G**, **H**). Data represent the mean ± standard error for three biological replicates. *p < 0.05, **p < 0.01, ***p < 0.001, ****p < 0.0001, ns: not significant.

We then asked whether ATAD2 overexpression plays a role in ovarian cancer growth and metastasis. We examined the effects of ATAD2 inhibition on ovarian cancer growth in a cell culture model using both pharmacological and genetic approaches. BAY-850 is a potent and isoform-selective small-molecule ATAD2 inhibitor with a 50% inhibitory concentration (IC_50_) of 166 nM. BAY-850 functions by preventing the binding of ATAD2 with acetylated histone [41]. We treated two ovarian cancer cell lines (PA-1 and SK-OV3) with various BAY-850 concentrations and assessed cell viability using 3-(4,5-dimethylthiazol-2-yl)-2,5-diphenyltetrazolium bromide (MTT) assays. BAY-850 treatment inhibited ovarian cancer cell viability in a dose-dependent manner (Fig. 2A). We also examined long-term survival of ovarian cancer cell using clonogenic assays and tumor-forming potential of ovarian cancer cells using a soft-agar assay [42]. BAY-850 treatment inhibited both colony-forming and tumor-forming ability of ovarian cancer cells in a dose-dependent manner (Fig. 2B–D). To further support our findings, we used two sequence-independent short hairpin RNA (shRNA) constructs to genetically knock down *ATAD2* expression and examined the effect of *ATAD2* knockdown on ovarian cancer growth using the soft-agar assay. Consistent with the results obtained with ATAD2 inhibitor BAY-850, we found that ATAD2 knockdown suppressed ovarian cancer tumor growth in the soft-agar assay (Supplementary Fig. 1A–D).

Analysis of patient-derived ovarian cancer samples revealed higher ATAD2 expression in metastatic ovarian cancer samples than in primary-site samples; therefore, we asked whether ATAD2 is necessary for the development of metastatic properties in ovarian cancer. We first performed Matrigel-based invasion assays and measured the invasive capabilities of ovarian cancer cells in the presence or absence of BAY-850. Compared with control-treated conditions, treatment with BAY-850 significantly inhibited the invasive potential of ovarian cancer cells (Fig. 2E, F). We also performed a wound-healing assay to assess the ability of ovarian cancer cells to migrate in the presence or absence of BAY-850. Treatment with BAY-850 significantly inhibited the ability of ovarian cancer cells to migrate (Fig. 2G–I) as compared with control-treated conditions. Collectively, these results establish that ATAD2 promotes ovarian cancer growth and metastasis in ovarian cancer cells.

### ATAD2 inhibition suppresses tumor growth and metastasis in complementary mouse models of ovarian cancer

**Fig. 3:**
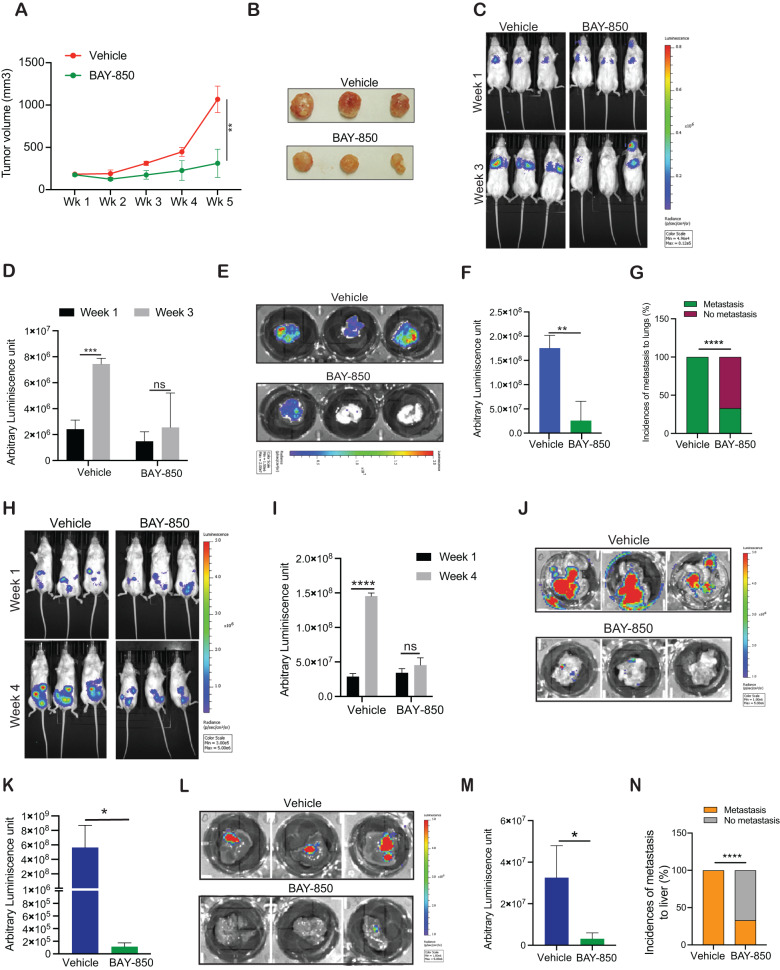
ATAD2 targeting inhibits ovarian cancer tumor growth and progression. **A** SK-OV3 cells were subcutaneously injected into the flanks of female NSG mice (*n* = 3). The mice were administered vehicle or BAY-850 (20 mg/kg body weight) intraperitoneally every other day. The average tumor volume was assessed weekly and plotted**. B** Representative images of tumors after 6 weeks of treatment with vehicle or BAY-850 shown in (**A**). **C**–**G** Firefly luciferase-labeled SK-OV3 cells were injected via the retroorbital route in female NSG mice (*n* = 3). The mice were administered vehicle or BAY-850 (20 mg/kg body weight) intraperitoneally every other day, and tumor growth was assessed by imaging the mice using IVIS imaging (**C**). Representative bioluminescence values from the mice at week 1 and 3 in vehicle or BAY-850 treatment (**D**). Bioluminescence images of lungs obtained from vehicle-treated or BAY-850-treated female NSG mice after 3 weeks of either vehicle or BAY-850 treatment (**E**). Relative luminescence values measured from lungs obtained from either vehicle-treated or BAY-850-treated NSG mice after 3 weeks of treatment (**F**). Percentage of mice with metastasis in lungs in vehicle or BAY-850 treated group (**G**). **H**–**N** Firefly luciferase-labeled SK-OV3 cells were intraperitoneally injected in female NSG mice (*n* = 3). The mice were administered vehicle or BAY-850 (20 mg/kg body weight) intraperitoneally every other day, and tumor growth was analyzed. Representative bioluminescence images 1 and 4 weeks after treatment with vehicle or BAY-850 (**H**). Relative luminescence value was measured 1 and 4 weeks after treatment with vehicle or BAY-850 (**I**). Bioluminescence images of intestines after 4 weeks after treatment in vehicle and BAY-850 treated group (**J**). Relative luminescence value in the intestine after 4 weeks of treatment with vehicle or BAY-850 (**K**). Representative bioluminescence images of liver after 4 weeks of treatment with vehicle or BAY-850 (**L**). Relative luminescence values from liver after 4 weeks of treatment with vehicle or BAY-850 (**M**). Percentage of mice with metastasis in liver in vehicle and BAY-850 treated group (**N**). Data represent the mean ± standard error for three biological replicates. *p < 0.05, **p < 0.01, ***p < 0.001, ****p < 0.0001, ns: not significant.

We next tested whether the ATAD2 inhibitor BAY-850 can suppress ovarian cancer growth in vivo. We first employed a xenograft mouse model, in which SK-OV3 ovarian cancer cells were subcutaneously injected into the flanks of female immunodeficient NSG mice. Mice were treated with vehicle or BAY-850, and subcutaneous tumor growth was measured. The results showed that BAY-850 treatment significantly suppressed subcutaneous tumor growth in mice compared with the vehicle treatment (Fig. 3A, B).

We next examined whether BAY-850 can inhibit ovarian cancer metastasis in vivo using lung metastasis-based mouse model of ovarian cancer metastasis. We first labeled SK-OV3 cells with the firefly luciferase gene (*F-Luc-*SK-OV3) and then retro-orbitally injected these cells into female NSG mice to induce lung metastasis. Mice were treated with vehicle or BAY-850, and bioluminescence imaging was used to monitor the metastatic growth in the lungs. We found that lung metastasis was significantly inhibited in BAY-850-treated mice compared with vehicle-treated mice (Fig. 3C–G). All three vehicle-treated mice (100%) developed lung metastasis, whereas only one of three BAY-850-treated mice (33.33%) developed lung metastasis (Fig. 3E–G).

Finally, we used a more stringent tumor growth and spontaneous metastasis mouse model, in which *F-Luc*-SK-OV3 and *F-Luc*-PA-1 cells were intraperitoneally injected into female immunodeficient NSG mice. Mice were treated with vehicle or BAY-850, and bioluminescence imaging was used to monitor tumor growth. BAY-850 treatment significantly suppressed intraperitoneal ovarian cancer tumor growth compared with vehicle treatment (Fig. 3H–K, Supplementary Fig. 2). We also measured spontaneous metastasis in this model and checked whether BAY-850 treatment prevented spontaneous liver metastasis from intraperitoneal (primary) site where tumors initially developed. Our result showed that spontaneous liver metastases of SK-OV3 ovarian cancer tumors were significantly reduced in the BAY-850-treated mice in comparison with control vehicle-treated mice (Fig. 3L, M). In the control group, all three mice (100%) showed liver metastasis, whereas in the BAY-850-treated group only one of three mice (33.33%) developed metastasis to the liver (Fig. 3N). These results demonstrate that ATAD2 inhibitor BAY-850 effectively suppresses both tumor growth and metastasis in multiple mouse models of ovarian cancer.

### ATAD2 inhibition promotes cell-cycle arrest and apoptosis

**Fig. 4:**
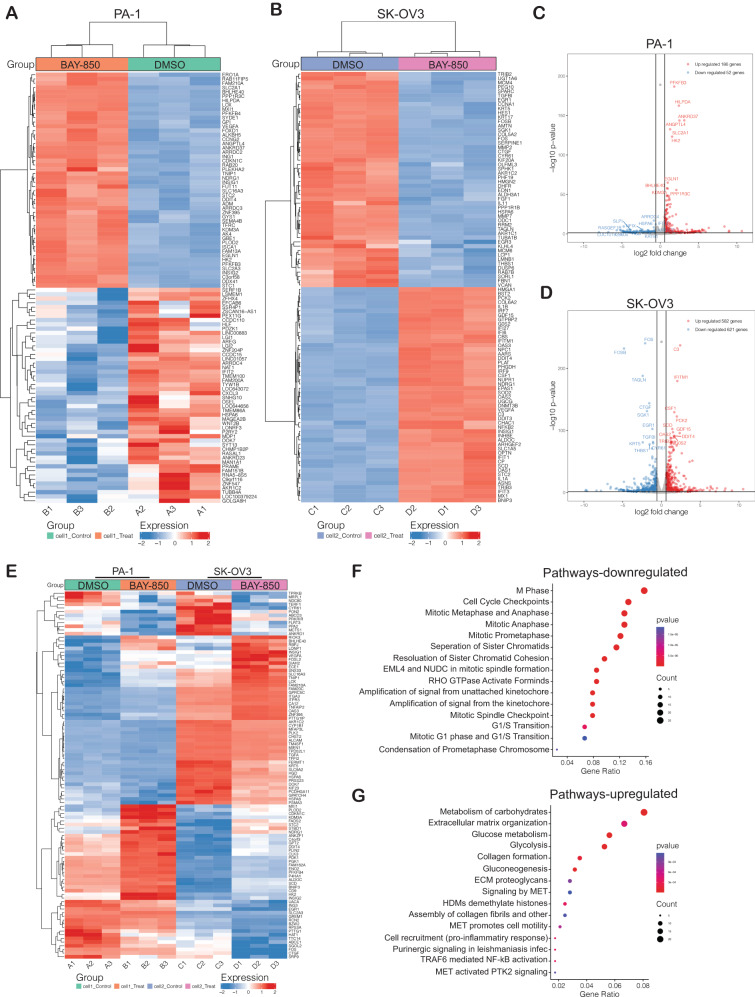
ATAD2 regulates functional pathways that affect ovarian cancer growth and proliferation. **A**, **B** The indicated ovarian cancer cell lines were treated with 5 µM BAY-850 for 48 h, and RNA sequencing was performed. Heatmaps show upregulated and downregulated genes (>1.5 fold) under BAY-850 treatment conditions relative to control DMSO treatment conditions. **C**, **D** Volcano plot showing upregulated and downregulated genes (>1.5 fold) in cells treated with 5 µM BAY-850 for 48 h relative control DMSO treated cells. **E** Heatmap showing genes that were significantly up- or down-regulated in both PA-1 and SK-OV3 cells treated with 5 µM BAY-850 for 48 h relative to control DMSO treated cells. **F**, **G** Pathways that were significantly activated or suppressed in both PA-1 and SK-OV3 cells following treatment with 5 µM BAY-850 for 48 h relative to cells treated with DMSO.

We next examined the mechanism by which ATAD2 promotes ovarian cancer growth. ATAD2 regulates transcription by modulating chromatin accessibility [43]. To identify its mechanism of action, we performed an RNA sequencing analysis of ovarian cancer cell lines (PA-1 and SK-OV3) following treatment with either vehicle (DMSO) or BAY-850. In PA-1 cells, BAY-850 treatment resulted in the significant (defined as ≥1.5 fold change in expression relative to vehicle treatment) downregulation of 52 genes and the significant upregulation of 186 genes compared with vehicle treatment (Fig. 4A, C, Supplementary Table 1). In SK-OV3 cells, BAY-850 treatment resulted in the significant downregulation of 621 genes and the significant upregulation of 582 genes compared with vehicle treatment (Fig. 4B, D, Supplementary Table 2). We then analyzed the RNA sequencing data to identify upregulated and downregulated genes common in both ovarian cancer cell lines (PA-1 and SK-OV3) following BAY-850 treatment. We found that in total 440 common genes were significantly (all genes with *p* value less than 0.05) upregulated and 253 common genes were significantly (all genes with p value less than 0.05) downregulated between the both the ovarian cancer cell lines (Supplementary Table 3). Using this information, we identified the top 100 genes altered in both ovarian cancer cell lines following BAY-850 treatment (Fig. 4E and Supplementary Table 4).

The functions of the genes identified as being altered in both ovarian cancer cell lines following BAY-850 treatment were investigated using biological pathway enrichment analysis (Supplementary Tables 5 and 6). Functional pathways that were significantly downregulated following BAY-850 treatment were associated with cell cycle, such as M phase, mitotic metaphase and anaphase, mitotic G1 phase, and the G1 to S transition (Fig. 4F, G, Supplementary Table 5). Many of these pathways have previously been shown to play an important roles in promoting both tumor growth and metastasis [44–46]. Thus, these results suggest that ATAD2 inhibition might regulate cell-cycle functions in ovarian cancer cells.

**Fig. 5:**
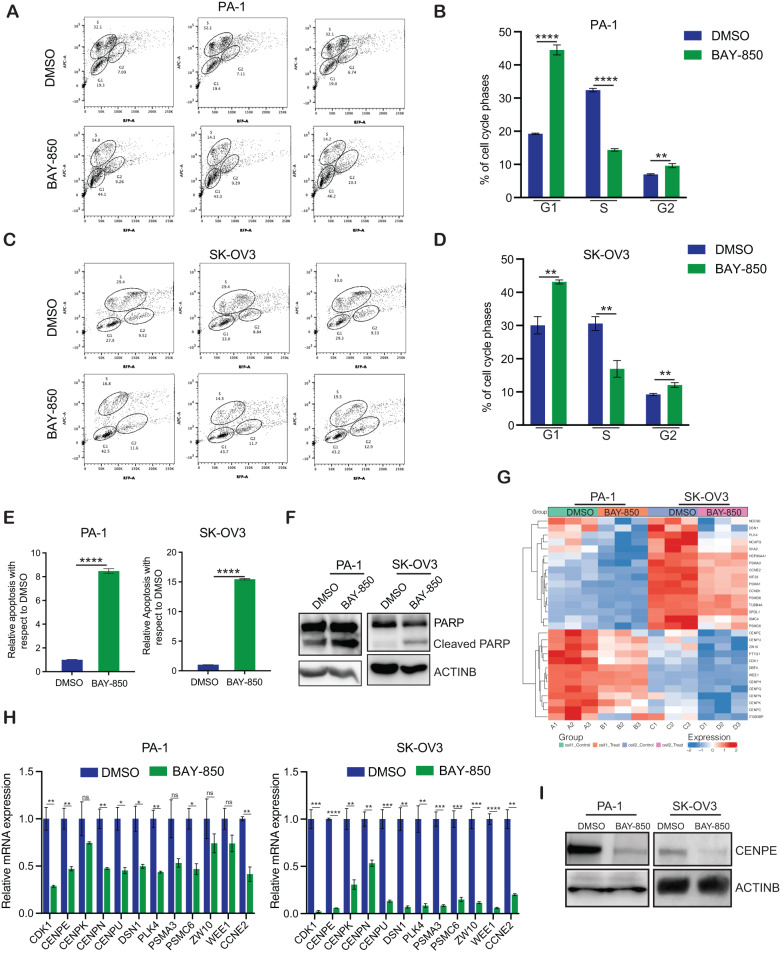
ATAD2 targeting results in cell-cycle arrest and apoptosis induction in ovarian cancer cells. **A and C** Flow cytometry analysis of ovarian cancer cells, PA-1 (A) and SK-OV3 (C), after treatment with DMSO or 5 µM BAY-850 for 48 h. **B and D** The percentage of cells in each phase of the cell cycle in PA-1 (B) and SK-OV3 (C) cells from (**A**, **C**). **E** The indicated ovarian cancer cell lines were treated with vehicle or 5 µM BAY-850 for 5 days, and apoptosis was measured via annexin V staining. Relative apoptosis in BAY-850-treated cells is plotted with respect to DMSO-treated cells. **F** The indicated ovarian cancer cell lines were treated with vehicle or 5 µM BAY-850 for 5 days, and poly (ADP-ribose) polymerase (PARP) cleavage was measured via immunoblotting. ACTINB was used as loading control. **G** Heatmaps showing alterations in centromere gene expression (based on RNA sequencing data) in both PA-1 and SK-OV3 cells treated with 5 µM BAY-850 for 48 h relative to gene expression in cells treated with DMSO. **H** Quantitative reverse-transcriptase-polymerase chain reaction (qRT-PCR) was used to measure RNA levels of selected centromere genes identified by RNA sequencing. Actin mRNA was used as the internal control. **I** The indicated ovarian cancer cell lines were treated with vehicle or 5 µM BAY-850 for 48 h, and centromere protein E (CENPE) protein levels were measured via immunoblotting. ACTINB was used as loading control. Data represent the mean ± standard error for three biological replicates. *p < 0.05, **p < 0.01, ***p < 0.001, ****p < 0.0001, ns: not significant.

Based on the results of biological- pathway enrichment analysis, we performed cell-cycle analyses of ovarian cancer cells in the presence and absence of BAY-850. Our cell-cycle analysis showed that BAY-850 treatment reduced the percentage of cells in S phase and increased the percentages of cells in the G1 and G2 phases compared with vehicle treatment (Fig. 5A–D). Prolonged cell-cycle defects can also induce apoptosis [47]. Therefore, we examined whether the BAY-850-induced disruption of the cell cycle translated into apoptosis induction in ovarian cancer cells by performing annexin V staining and examining PARP cleavage to monitor apoptosis. We found that BAY-850 treatment of ovarian cancer cells increased the numbers of annexin V-positive cells and increased PARP cleavage compared with vehicle treatment (Fig. 5E, F). Collectively, these results demonstrate that ATAD2 inhibition via BAY-850 leads to cell-cycle arrest and apoptosis induction, which, in turn, suppresses ovarian cancer growth and progression.

### ATAD2 regulates the expression of centromeric protein CENPE

To gain mechanistic insight regarding how ATAD2 inhibition results in cell-cycle arrest and apoptosis induction, we analyzed RNA sequencing data to identify the genes associated with cell-cycle functions that are altered in ovarian cancer cells following BAY-850 treatment. In both the PA-1 and SK-OV3 cells, treatment with BAY-850 resulted in the downregulation of multiple genes that encode centromeric proteins (CENPs; Fig. 5G). CENPs play important roles in centromere function and mitosis and can regulate tumor cell proliferation [48, 49]. CENPs are also associated with responses to cancer therapy and may affect patient survival [50, 51]. We first validated the RNA sequencing results by performing quantitative reverse transcriptase-polymerase chain reaction (qRT-PCR) and immunoblotting. Our results indicate that CENPE was significantly downregulated in both ovarian cancer cell lines at both the mRNA and protein levels following BAY-850 treatment (Fig. 5H, I, Supplementary Fig. 3).

**Fig. 6:**
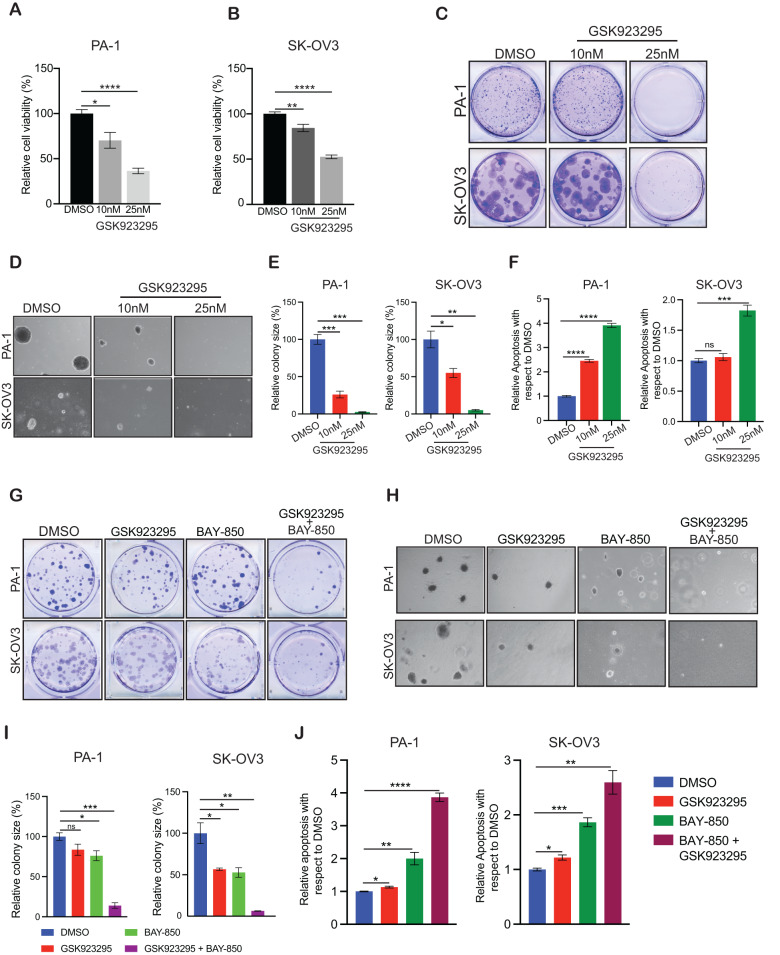
ATAD2 co-targeting with CENPE cause potent ovarian cancer tumor growth inhibition. **A**, **B** The indicated ovarian cancer cell lines were treated with various concentrations of GSK923295 for 3 days, and survival was assessed by 3-(4,5-dimethylthiazol-2-yl)-2,5-diphenyltetrazolium bromide (MTT) assay. Cell survival is presented relative to the survival of DMSO-treated cells. **C** The indicated ovarian cancer cell lines were treated with indicated concentrations of GSK923295 for 2–4 weeks. Cell survival was measured using clonogenic assays. Representative images are shown. **D** The indicated ovarian cancer cell lines were treated with indicated concentrations of GSK923295and analyzed for their abilities to grow in soft-agar assays. Representative images are shown; scale bar, 500 µm. **E** Relative colony sizes for the images shown in (**D**). **F** The indicated ovarian cancer cell lines were treated with vehicle or indicated concentrations of GSK923295 for 48 h, and apoptosis was measured via annexin V staining. Apoptosis in GSK923295-treated cells is presented relative to apoptosis in DMSO-treated cells. **G** PA-1 ovarian cancer cell line was treated with DMSO, 0.2 µM BAY-850 alone, 10 nM GSK923295 alone, or both in combination for 2–4 weeks and SK-OV3 ovarian cancer cell line was treated with DMSO, 1 µM BAY-850 alone, 15 nM GSK923295 alone, or both in combination for 2–4 weeks. Cell survival was measured in clonogenic assays. Representative images are shown. **H** PA-1 ovarian cancer cell line was treated with DMSO, 0.2 µM BAY-850 alone, 10 nM GSK923295 alone, or both in combination and SK-OV3 ovarian cancer cell line was treated with DMSO, 1 µM BAY-850 alone, 15 nM GSK923295 alone, or both in and analyzed for their abilities to grow in soft-agar assays. Representative images are shown; scale bar, 500 µm. **I** Relative colony sizes for the images shown in (**H**). **J** PA-1 ovarian cancer cell line was treated with DMSO, 0.2 µM BAY-850 alone, 10 nM GSK923295 alone, or both in combination and SK-OV3 ovarian cancer cell line was treated with DMSO, 1 µM BAY-850 alone, 15 nM GSK923295 alone, or both in combination for 48 h and apoptosis was measured via annexin V staining. Relative apoptosis in treated cells is plotted with respect to DMSO-treated cells. Data represent the mean ± standard error for three biological replicates. *p < 0.05, **p < 0.01, ***p < 0.001, ****p < 0.0001, ns: not significant.

CENPE is a microtubule plus-end-directed kinetochore motor protein, and its loss prevents chromosome alignment, inhibits the attachment of microtubules to kinetochores, and induces mitotic arrest during prometaphase and metaphase [52, 53]. CENPE is also associated with various cancer types, and its loss results in cell division defects and cell death. Several CENPE inhibitors have been developed for cancer treatment [54, 55], including GSK923295, which is currently being studied in a phase I clinical trial of adult patients with solid tumors that have not responded to common therapies [56]. To examine the role played by CENPE in ovarian cancer growth, we treated ovarian cancer cell lines with GSK923295, a potent CENPE inhibitor with an inhibitory constant (K_i_) of 3.2 nM [55]. We first examined whether GSK923295 treatment affected ovarian cancer cell growth using MTT, clonogenic and soft-agar assays. We found that GSK923295 treatment inhibited the ovarian cancer cell viability in MTT assay, colony-forming ability and soft-agar growth of ovarian cancer cells in a concentration-dependent manner (Fig. 6A-E). We next examined the effect of GSK923295 treatment on apoptosis induction and found that GSK923295 treatment induced apoptosis as demonstrated by increased numbers of annexin V-positive ovarian cancer cells as compared to control-treated cells (Fig. 6F). In sum, these results demonstrate that CENPE inhibition phenocopied ATAD2 inhibition effects in suppressing ovarian cancer tumor growth.

Based on these results, we tested the combined effects of treatment with both GSK923295 and BAY-850 on ovarian cancer cell growth. We observed that combined treatment with GSK923295 and BAY-850 at suboptimal doses resulted in stronger ovarian cancer tumor growth inhibition than either treatment alone, as assessed by both clonogenic and soft-agar assays (Fig. 6G–I). Further, combined treatment with both GSK923295 and BAY-850 led to enhanced apoptosis induction in ovarian cancer (Fig. 6J) compared with either treatment alone. Collectively, these results identify ATAD2 as a novel driver of ovarian cancer cell growth and metastasis that functions via CENPE to prevent cell cycle arrest and apoptosis and suggest that ATAD2 can be targeted either alone or in combination with CENPE inhibitor to provide therapeutic benefits to ovarian cancer patients.

## Discussion

**Fig. 7:**
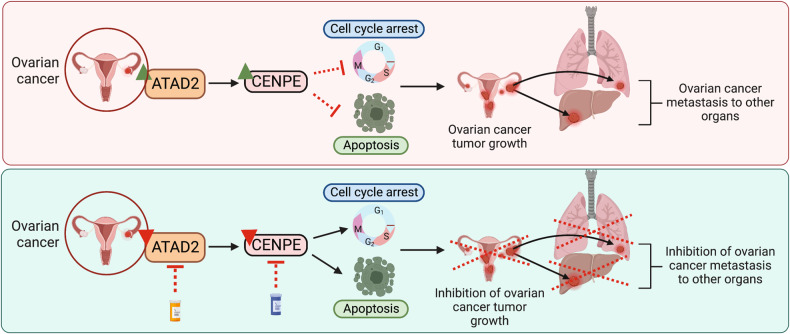
Model of therapeutic ATAD2 inhibition. ATPase family AAA domain-containing 2 (ATAD2) promotes ovarian cancer tumor growth and progression by regulating the expression of centromere protein E (CENPE), which prevents cycle arrest and apoptosis. ATAD2 and CENPE can both be targeted via small-molecule inhibitors, either alone or in combination, to provide effective ovarian cancer therapy.

Ovarian cancer is a leading cause of cancer-related death among women [2]. Standard treatments for newly diagnosed cancer consist of cytoreductive surgery and platinum-based chemotherapy [4]. Additionally, anti-angiogenic agents, poly (ADP-ribose) polymerase (PARP) inhibitors, and immunological therapies are used to treat ovarian cancer [5]. Despite aggressive therapy, the 5-year survival rate among women diagnosed with advanced-stage ovarian cancer is only 31%, making ovarian cancer one of the most lethal gynecological malignancies [57]. Therefore, identifying potential new drivers and drug targets in ovarian cancer has become crucial. In this study, we found that ATAD2 inhibition suppressed the tumor growth and metastasis of ovarian cancer cells. Furthermore, we observed that the CENPE inhibitor GSK923295 significantly potentiated the tumor-suppressive effects of the ATAD2 inhibitor BAY-850 in ovarian cancer cells. Collectively, our findings suggest that the administration of BAY-850 together with GSK923295 might represent a new therapeutic approach for ovarian cancer. The study results are summarized in Fig. 7 and discussed below.

In the majority of ovarian cancer cases ~95%, p53 is inactivated due to either mutations or genetic deletion [39]. In our study, we used cell lines that harbor different types of p53 mutation. In SK-OV3 cells p53 is deleted [40] and in PA-1 cells p53 is mutated [58]. We observed that ATAD2 inhibitor BAY-850 successfully inhibited the growth of both the cell lines although with different sensitivity. We anticipate that the difference in sensitivity could be due to the other factors beyond ATAD2 that could modulate the intrinsic sensitivity of ovarian cancer cells to ATAD2 inhibitors. Hence, identifying such factors will represent an important future direction for our studies.

ATAD2 is overexpressed in many different cancer types [17–23] and plays a role in tumor development. ATAD2 acts as an epigenetic reader, transcription factor, and co-activator involved in diverse signaling pathways, such as the Rb-E2F-c-Myc pathway, the p53- and p38-MAPK-mediated apoptotic pathway, and the hedgehog signaling pathway [25, 26, 59]. Therefore, ATAD2 serves as a cancer biomarker and therapeutic target. Based on the important roles played by ATAD2 in multiple cancer types, efforts have been focused on developing a new class of potent and specific ATAD2 inhibitors that target its bromodomain, and these inhibitors are currently being tested as therapies for various cancer types [60–62].

In our study, we show that ATAD2 is overexpressed and promotes tumor growth and metastasis in ovarian cancer models. In a previous study, the ATAD2 yeast homolog Yta7 was found to function as a deposition factor for CENPA at yeast centromeres. This prior study further showed that Yta7 acts as a hexameric AAA^+^ ATPase that unfolds CENPA/H4 and delivers Scm3/HJURP for incorporation into the centromeric nucleosome, and defects in this process lead to kinetochore instability and chromosome segregation defects [63]. Our study, for the first time, shows that ATAD2 is a promoter of ovarian cancer tumor growth by regulating CENPE expression.

CENPE is a microtubule plus-end-directed kinetochore motor protein that plays crucial roles in chromosome congression, the capture of spindle microtubules at kinetochores, spindle assembly checkpoints, chromosome alignment, and segregation [64, 65]. CENPE inhibition prevents chromosome alignment, inhibits the attachment of microtubules to kinetochores, and induces cell-cycle (mitotic) arrest [52, 53]. CENPE is also associated with various cancer types [66–68], and multiple CENPE inhibitors have been developed [54, 55] as potential cancer therapeutics. One of the most successful CENPE inhibitors is GSK923295, an allosteric inhibitor that binds the ATPase pocket [55], which shows broad antitumor activity in preclinical in vivo models [69, 70]. Phase I clinical studies performed in adult patients with solid tumors that have not responded to common therapies demonstrated a dose-proportional pharmacokinetic with mild adverse effects (NCT00504790) [56]. Based on our results, together with the findings of previous studies and clinical trials, CENPE inhibitors can be rapidly utilized in clinical settings as an effective potential ovarian cancer therapy. Additionally, our results indicate that CENPE inhibitors can be combined with ATAD2 inhibitors for effective ovarian cancer therapy (Fig. 7).

Various clinical antibody treatments have been developed targeting programmed cell death protein 1 (PD-1) and its ligand (PD-L1), such as pembrolizumab. These treatments inhibit immune checkpoints and increase T-cell-based clearance of tumor cells [71]. Despite the success of these therapies in various cancer types, such as melanoma, only a small percentage of ovarian cancer patients derived benefits from these therapies in large clinical trials (JAVELIN 100 (NCT02718417), JAVELIN 200 (NCT0280058), and IMagyn050 (NCT03038100)). Therefore, based on our results, ATAD2 inhibitors, combined with CENPE inhibitors or other immunotherapeutic drugs, such as pembrolizumab, should be explored as potentially effective ovarian cancer therapies in patients with advanced or metastatic disease who continue to lack effective and durable therapeutic options. These results also support the need for the clinical testing of ATAD2 inhibitors combined with CENPE inhibitors for treating ovarian cancer patients.

## Materials and methods

### Cell-culture conditions and reagents

Short tandem repeat (STR) profile verified Ovarian cancer cell lines (PA-1 and SK-OV3) and HEK-293T cells were purchased American Type Culture Collection (ATCC, Manassas, VA, USA) and maintained as recommended by the ATCC. PA-1 cells were grown in Dulbecco’s Modified Eagle Medium (DMEM; Life Technologies, Thermo Fisher Scientific, Waltham, MA, USA), supplemented with 10% fetal bovine serum (FBS; Life Technologies, Thermo Fisher Scientific) and 1% penicillin/streptomycin (Life Technologies) under 5% CO_2_. SK-OV3 cells were grown in Roswell Park Memorial Institute (RPMI) 1640 medium (Life Technologies, Thermo Fisher Scientific) supplemented with 10% FBS and 1% penicillin/streptomycin in 5% CO_2_. Mycoplasma negative status for all cell lines was verified using MycoAlert mycoplasma detection kit (Lonza), and were routinely tested for the lack of mycoplasma contamination.

### shRNA, lentivirus preparation, and stable cell line generation

Gene-specific *ATAD2* shRNAs were obtained from the Open Biosystems. The catalog numbers for the shRNAs are provided in Supplementary Table 7. For lentivirus production, plasmids were transfected into HEK-293T cells along with the PDM2.G and pSPAX2 packaging plasmids. After 48 h, the lentivirus/retrovirus-containing supernatants were harvested, filtered, and used for infections. Lentiviral shRNA-infected SK-OV3 cells were selected using 0.5 μg/mL puromycin.

### Chemical inhibitors

BAY-850 (Cat. No.: HY-119254) and CENPE inhibitor (Cat. No.: GSK923295) and were purchased from Selleck Chemical LLC, and dissolved for cell culture and in vivo experiments as suggested in the data sheet. Relevant information is provided in Table S7. The treatment conditions are described in the corresponding figure legends.

### ATAD2 mRNA expression analysis of patient-derived ovarian cancer patient samples

Datasets of gene expression in ovarian cancer and normal skin samples were identified by a search of the Oncomine cancer profiling database. The TCGA ovarian [33] Yoshihara ovarian [34], Anglesio ovarian [35], Tothill ovarian [36], Meyniel ovarian [37], Lu ovarian [38] datasets were used for analysis.

### ATAD2 protein expression analysis of patient-derived ovarian cancer samples from the Human Tissue Atlas Dataset Using immunohistochemistry (IHC)

The Human Protein Atlas is a publicly available database containing millions of high-resolution images showing the spatial distribution of proteins detected with 15,598 different antibodies (release 9.0, November 2011) in 46 different normal human tissue types and 20 different cancer types, as well as 47 different human cell lines. Samples containing normal and cancerous tissue were collected and paraffin-embedded following approval by the local ethics committee. Each antibody listed in the database was used for IHC staining of both normal and cancerous tissue.

### Transcription-factor analysis using PROMO

Transcription factor binding with 100% sequence identity on the promoter region of ATAD2 was identified using the PROMO tool (http://alggen.lsi.upc.es/cgi-bin/promo_v3/promo/promoinit.cgi?dirDB=TF_8.3) [72]. We s*e*lected a 2-kB upstream promoter region of the ATAD2 gene to find the human transcription factors that bound using PROMO.

### 4,5-dimethylthiazol-2-yl)-2,5-diphenyltetrazolium bromide (MTT) assay

For MTT assay, 2 × 10^3^ of PA-1 and SK-OV3 cells were plated in a 100 µl volume in 96-well plates. After 24 h, BAY-850 inhibitor, used at a range of concentrations (0.1 μM, 0.2 μM, 0.5 μM, 1 μM, 2 μM, 5 μM) and CENPE inhibitor GSK923295 (10 nM, 25 nM) was mixed in 100 μl of medium and added to the cells. After 3 days of inhibitor treatment, the cell viability was evaluated. To do this, 20 µl of 5 mg/ml MTT solution dissolved in 1× PBS was added to each well and incubated for 2 h at 37 °C incubator. The MTT (1-(4,5-Dimethylthiazol-2-yl)-3,5-diphenylformazan) solution was removed gently, and 100 µl of DMSO were added. After mixing well by pipetting, absorbance was measured at 590 and 630 nm. An average was calculated for both readings, and then measurement at 630 nm was subtracted from that at 590 nm. The relative cell viability was plotted with respect to control DMSO-treated cells.

### Clonogenic assay

For clonogenic assay, PA-1 and SK-OV3 cells were seeded in a six-well plate at 1 × 10^3^ numbers. Cells were seeded in triplicate wells of a 6-well plate and incubated for 24 h, at which time the cells were treated with vehicle or inhibitor. After 3–4 weeks, colonies were fixed using a fixing solution containing 50% methanol and 10% acetic acid and then stained with 0.05% Coomassie blue (Sigma-Aldrich, St. Louis, MO, USA). Representative images each sample under the indicated conditions is shown.

### Soft-agar assay

Soft-agar assays were performed by seeding 7 × 10^3^ PA-1, and SK-OV3 cells onto 0.4% low-melting-point agarose (Sigma-Aldrich) layered on top of 0.8% agarose. After 3–6 weeks of incubation, colonies were stained with a 0.05% crystal violet solution and imaged using a microscope. Colony size was measured using microscopy and ImageJ software (https://imagej.nih.gov/ij/) and plotted as the percent relative colony size compared with control cells. Statistical analysis was performed using Student’s t-tests in the GraphPad Prism 7 software.

### Matrigel-invasion assay

Invasion assays were performed in BioCoat Growth Factor Reduced Matrigel Invasion Chambers (Cat#354483, BD Biosciences, Franklin Lakes, NY, USA) using ovarian cancer cells, and these Cells were treated with vehicle or inhibitor and serum-starved for 6 h and then seeded in triplicate into the top chamber at a density of 5 × 10^4^ cells (PA-1 & SK-OV3)/insert and 1 × 10^4^ cells (PA-1 & SK-OV3)/insert in low-serum medium (0.2% FBS). The cells were incubated for 24 h to allow invasion toward the serum-rich medium (10% FBS) in the lower chamber, where the vehicle or inhibitors was added in culture media both upper and lower chambers. The number of cells invading the Matrigel was quantified by DAPI staining and imaging; 8–12 fields per membrane were counted, and nuclei quantification was performed using ImageJ software. (https://imagej.nih.gov/ij/).

### Wound healing assay

Ovarian cancer cell lines PA-1 and SK-OV3 were seeded in 6 well plate at a density of 2 × 10^5^ cells per well and grown plates until fully confluent. A scratch was then created using a sterile 20 μl pipette tip, and the cell were then treated with 5 µM of BAY-850. Cell migration into the wound was monitored at 0, 24, and 48 h using light microscopy. Quantification of wound healing was performed using ImageJ software (https://imagej.nih.gov/ij/).

### RNA sequencing and data analysis

Ovarian cancer cell lines (PA-1 and SK-OV3 cells) were treated with BAY-850 (5 μM) and control for 48 h were used to prepare total RNA for gene-expression analysis on an Illumina HiSeq 2500 system. Total RNA was extracted using TRIzol^®^ reagent (Invitrogen) according to the manufacturer’s instructions and purified on RNAeasy mini columns (Qiagen) according to the manufacturer’s instructions. Then, mRNA was purified from approximately 300 ng total RNA using oligo-dT beads and sheared by incubation at 94 °C. Following first-strand synthesis with random primers, second-strand synthesis was performed with dUTP to generate strand-specific libraries. The cDNA libraries were then end-repaired and A-tailed. Adapters were ligated, and second-strand digestion was performed using Uracil-DNA-Glycosylase. Indexed libraries that met appropriate cutoffs for both were quantified by qRT-PCR using a commercially available kit (KAPA Biosystems, Wilmington, MA, USA). The insert-size distribution was determined using LabChip GX or an Agilent Bioanalyzer. Samples with a yield ≥ 0.5 ng/μl were used for sequencing on the Illumina HiSeq 2500 system. Images were converted into nucleotide sequences by the base-calling pipeline RTA 1.18.64.0 and stored in FASTQ format.

### RNA preparation, cDNA preparation, and quantitative PCR analysis

Total RNA was extracted with TRIzol Reagent (Invitrogen) and purified using the RNeasy Mini Kit (Qiagen); cDNA was generated using the M-MuLV First Strand cDNA Synthesis Kit (New England Biolabs) according to the manufacturer’s instructions. Quantitative RT-PCR was performed with gene-specific primers, using the Power SYBR-Green Master Mix (Applied Biosystems) according to the manufacturer’s instructions. Actin was used as an internal control. Primer sequences are provided in Supplementary Table 7.

### CUT&RUN assay

CUT&RUN assays were performed with SK-OV3 cells using the CUT&RUN Assay Kit (Cat#86652; Cell Signaling Technology Danvers, MA, USA) according to the manufacturer’s instructions. Briefly, 2 × 10^5^cells were harvested, washed, bound to activated Concanavalin A‐coated magnetic beads, and permeabilized. The bead-cell complexes were incubated overnight with the appropriate antibody at 4 °C. Then, the complexes were washed three times, and the cells were resuspended in 100 μl pAG/MNase and incubated for 1 h at room temperature. The samples were then washed three times with digitonin buffer with protease inhibitors, resuspended in 150 μl digitonin buffer, and incubated 5 min on ice. MNase was activated by adding calcium chloride, and the samples were incubated at 4 °C for 30 min. The reaction was stopped by adding 150 μl stop buffer, and the samples were incubated at 37 °C for 10 min to release the DNA fragments. The DNA was extracted using the DNA purification columns included in the CUT&RUN Assay Kit. qPCR was then performed using ATAD2 promoter-specific primers, and relative fold-change was calculated as the ratio of immunoprecipitated DNA to IgG-precipitated DNA. The primer sequences and antibodies used for the CUT&RUN assays are listed in Supplementary Table 7.

### Immunoblotting analysis

Whole-cell protein extracts were prepared using RIPA lysis buffer (Pierce) containing Protease Inhibitor Cocktail (Roche) and Phosphatase Inhibitor Cocktail (Sigma-Aldrich, St. Louis, MO). Lysed samples were centrifuged at 12,000 rpm for 40 min, and clarified supernatants were stored at −80 °C. Protein concentrations were determined using Bradford Protein Assay Reagent (Bio-Rad Laboratories, Hercules, CA, USA). Equal amounts of protein samples were electrophoresed on 10% or 12% sodium dodecyl sulfate (SDS)- polyacrylamide gels and transferred onto polyvinylidene difluoride (PVDF) membranes (Millipore, Burlington, MA, USA) using a wet-transfer apparatus from Bio-Rad. The membranes were blocked with 5% skim milk and probed with primary antibodies in 5% BSA. After washing, the membranes were incubated with the appropriate horseradish peroxidase (HRP)-conjugated secondary antibodies (1:2,000) (GE Healthcare Life Sciences, Marlborough, MA, USA). The blots were developed using SuperSignal West Pico or Femto Chemiluminescent Substrate (Thermo Fisher Scientific). All antibodies used for immunoblotting are listed in Supplementary Table 7.

### Flow-cytometry analysis (FACS)

Flow cytometry analysis in PA-1 and SK-OV3 cells were measured by Click-iT EdU flow cytometry assay kit (Invitrogen). EdU (5-ethynyl-2´-deoxyuridine), a thymidine analog which incorporated during DNA replication. EdU (10 μM) was added to PA-1 and SK-OV3 cells and incubated (for PA-1 2 h and SK-OV3 12 h) in humidified CO_2_ incubator at 37 °C. Cells were trypsinised and rinsed with 3 ml of PBS with 1% BSA and then cell pellet fixed with 100 μL of Click-iT™ fixative (Component D). After 15 mins of incubation at room temperature in dark, wash the cells with 3 mL of 1% BSA in PBS and pellet the cells. Cells were premetallized with the 100 μL of 1X Click-iT™ permeabilization & wash reagent and incubated for 15 mins. The cell pellet washed with the PBS with 1% BSA and incubated with the Click-iT™ Plus reaction cocktail (containing Alexa Fluor 488) for 30 mins at room temperature. Wash the cells once with 3 mL of 1X Click-iT™ permeabilization and wash reagent, centrifuge the cells, and remove the supernatant. DNA Ribonuclease A and propidium iodide mixture were added to stain the DNA then the Samples were analyzed by traditional flow cytometer.

### Apoptosis measurement using annexin V/propidium iodide staining

Annexin V binding to cells was measured with the use of an Annexin V staining kit (BD PharmingenTM #556547, BD Pharmingen, San Diego, CA, USA) according to the manufacturer’s protocol. In brief, PA-1 or SK-OV3 cells were treated with vehicle or inhibitor for 48 h. After treatment, cells were collected, washed twice with 1× PBS and resuspended in 1× Binding buffer and stained with 5 µL FITC-Annexin V and 5 µL of PI and incubated for 15 min in the dark. After incubation, cells were analyzed with FACS using LSR Fortessa (BD Biosciences, Franklin Lakes, NJ, USA).

### Subcutaneous xenograft-based mouse tumorigenesis experiment with BAY-850 treatment

SK-OV3 (5 × 10^6^) cells in 100 µl mixed with 100 µl of matrigel were injected subcutaneously into 5–6-week-old female NSG mice (stock No. 005557). Tumor volume was measured every week, and tumor size was calculated using the following formula: length × width^2^ × 0.5. When the tumor volumes reached ∼80–100 mm^3^, the mice were treated with either vehicle (0.5% methyl cellulose in water) or BAY-850 (20 mg/kg body weight) intraperitoneally every other day until the end of the experimental period. Tumor volume was measured every week and plotted. Subcutaneous tumors from individual groups were harvested and imaged. All protocols for mouse experiments were approved by the Institutional Animal Care and Use Committee of the University of Alabama at Birmingham (UAB).

### Retroorbital-based lung metastases mouse tumorigenesis experiment

SK-OV3 cells stably expressing firefly luciferase under the control of a cytomegalovirus promoter were generated by co-transfection of the transposon vector piggyBac GFP-Luc and the helper plasmid Act-PBase as described previously [73]. Cells with stable transposon integration were selected using blasticidin S (Thermo Fisher Scientific). SK-OV3 GFP-Luc cells (500,000) were then injected retro-orbitally into female 5–6-week-old NSG mice (Jackson Laboratory, Stock No. 005557). For monitoring of lung metastasis, imaging was performed every week using the IVIS Spectrum In Vivo Imaging System (Perkin Elmer, Waltham, MA, USA). When the tumors were palpable, mice were treated with either vehicle (0.5% methyl cellulose in water) or BAY-850 (20 mg/kg body weight) intraperitoneally every other day until the end of the experimental period. Total luminescence counts of the tumor-bearing areas were measured using the Living Image in vivo imaging software (Perkin Elmer). At the end of the experiment (4 weeks after the start of treatment), the mice were sacrificed, images of the tumors were captured, and the lungs were imaged using the IVIS Spectrum (Perkin Elmer). All protocols were approved by the UAB Institutional Animal Care and Use Committee.

### Intraperitoneal-based tumor growth and metastases mouse tumorigenesis experiment

SK-OV3 and PA-1 cells stably expressing firefly luciferase were injected intraperitoneally into female 5–6-week-old NSG mice (Jackson Laboratory, Stock No. 005557). For monitoring of tumor growth and metastasis, imaging was performed every week using the IVIS Spectrum In Vivo Imaging System (Perkin Elmer, Waltham, MA, USA). When the tumors were palpable, mice were treated with vehicle (0.5% methyl cellulose in water) or BAY-850 (20 mg/kg body weight) intraperitoneally every other day until the end of the experimental period. Total luminescence counts of the tumor-bearing areas were measured using the Living Image in vivo imaging software (Perkin Elmer). At the end of the experiment (4 weeks after the start of treatment), the mice were sacrificed, images of the intestine and liver tumors were captured using the IVIS Spectrum (Perkin Elmer). All protocols were approved by the UAB Institutional Animal Care and Use Committee.

### Statistical analysis

All experiments were conducted with at least three biological replicates. Results for individual experiments are expressed as mean ± standard error of the mean (SEM). For animal experiments, the sample size was chosen based on the preliminary experiments and previous experience with similar studies. Also, no blinding was done for the animal experiments. For the analysis of tumor progression in mice, the statistical assessment was performed using the area under the curve (AUC) method on GraphPad Prism, version 9.0 for Macintosh (GraphPad Software, San Diego, CA, USA; www.graphpad.com). The *P*-values for the rest of the experiments were calculated using the two-tailed unpaired Student’s t-test in GraphPad Prism version 9.0 for Macintosh (GraphPad Software). For analyzing the incidences of spontaneous metastasis to lungs or liver, the contingency analysis was performed using chi-square test in GraphPad Prism version 9.0 for Macintosh (GraphPad Software, San Diego, California, USA).

## Supplementary information

Supplementary Figures and Tables

Supplementary Table 1

Supplementary Table 2

Supplementary Table 3

Supplementary Table 4

Supplementary Table 5

Supplementary Table 6

Supplementary Table 7

Original Data File

Reproducibility checklist

## Data Availability

All datasets generated and analyzed during the study are included in this published article and its Supplementary Information files. Additional data are available from the corresponding author on reasonable request.
