# Supplementary material for: ATAD2 is a driver and a therapeutic target in ovarian cancer that functions by upregulating CENPE
Source: Cell Death Dis. 2023 Jul 21;14(7):456. doi: 10.1038/s41419-023-05993-9 (PMC10362061; doi:10.1038/s41419-023-05993-9)
Supplement: Supplementary file 1 — Supplementary Figures and Tables [file 41419_2023_5993_MOESM1_ESM.pdf]

SUPPLEMENTARY FIGURES and LEGENDS

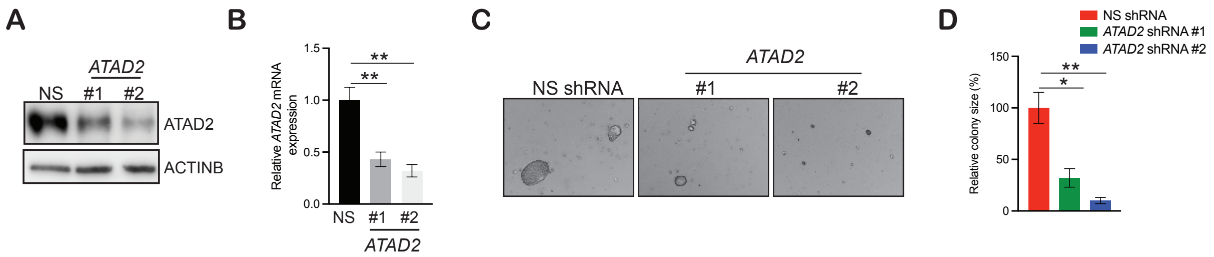

Supplementary Fig. 1. ATAD2 knockdown inhibits the growth of ovarian cancer cells. **A.** Immunoblotting of ATAD2 in SK-OV3 cell lines expressing *ATAD2* or NS shRNA. ACTINB protein was measured as loading controls. **B.** SK-OV3 cell lines expressing either *ATAD2* or NS shRNA were analyzed for *ATAD2* expression by RT-qPCR. *ATAD2* mRNA expression in *ATAD2* shRNA-expressing cells relative to NS shRNA expressing cells is shown. ACTB was used for normalization. **C.** The indicated SK-OV3 cell lines expressing either *ATAD2* or NS shRNA were analyzed in the soft agar assay. Representative images of soft agar colony formation are shown**.** Scale bar, 500 μm. **D.** Relative soft agar colony size for the data shown in panel C. Data represent the mean ± standard error of three biological replicates. *p<0.05, ** p<0.01.

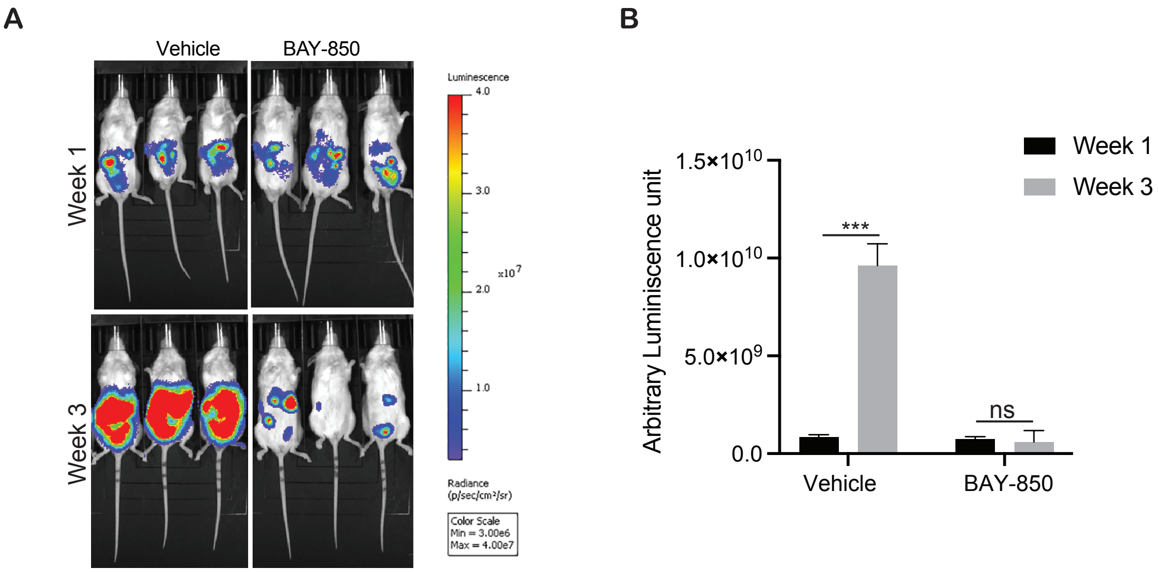

Supplementary Fig. 2. ATAD2 targeting inhibits ovarian cancer tumor growth.

A. Firefly luciferase–labeled PA-1 cells were intraperitoneally injected in female NSG mice (n=3). The mice were administered vehicle or BAY-850 (20 mg/kg body weight) intraperitoneally every other day, and tumor growth was analyzed. Representative bioluminescence images 1 and 3 weeks after treatment with vehicle or BAY-850. **B**. Relative luminescence value was measured 1 and 3 weeks after treatment with vehicle or BAY-850. Data represent the mean ± standard error for three biological replicates. ***p<0.001 and ns: not significant

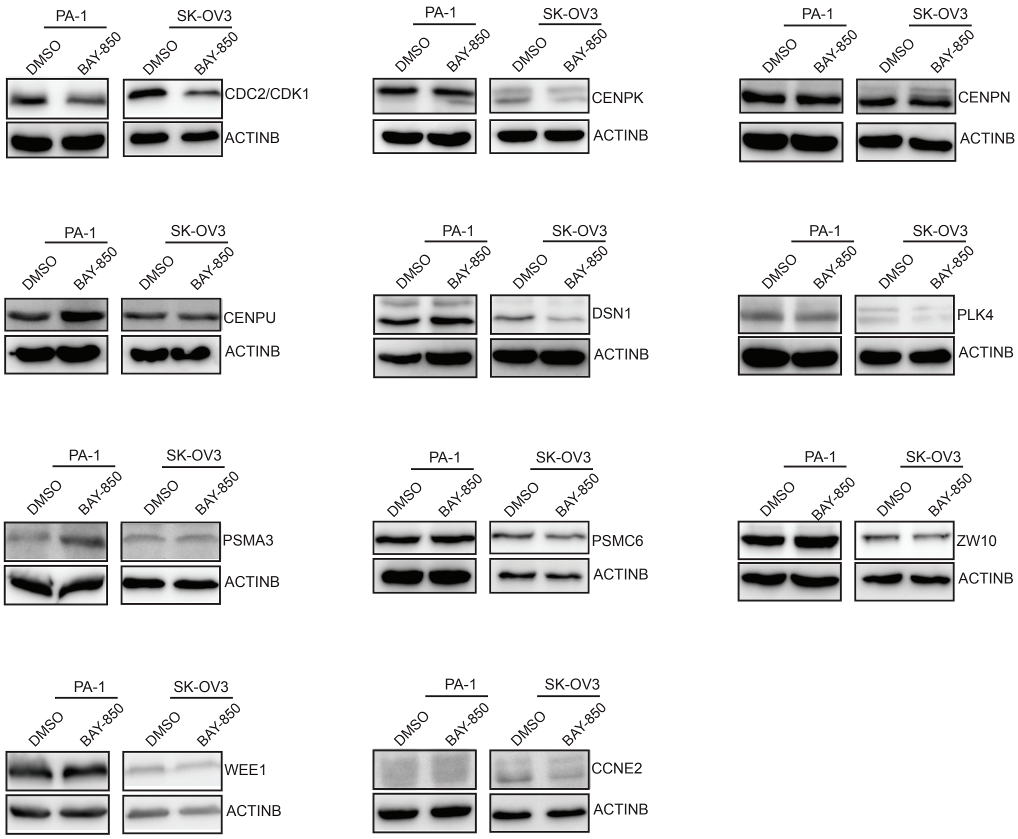

Supplementary Fig. 3. Effect of ATAD2 inhibitor BAY-850 on the expression of centromeric proteins. Immunoblotting of the indicated proteins in PA-1 and SK-OV3 cell lines treated with vehicle or 5 μM BAY-850 for 48 h. ACTINB protein was measured as loading controls.

SUPPLEMENTARY TABLES

Supplementary Table 1: Significant differentially expressed genes in BAY-850 treated PA-1 cells. RNA sequencing data showing significant differentially expressed gene in PA-1 cell upon treatment with ATAD2 inhibitor BAY-850 5µM for 48 h in comparison to control DMSO treated cells. Genes upregulated and downregulated ≥1.5-fold is presented.

Supplementary Table 2: Significant differentially expressed genes in BAY-850 treated SK-OV3 cells. RNA sequencing data showing significant differentially expressed gene in SK-OV3 cell upon treatment with ATAD2 inhibitor BAY-850 5µM for 48 h in comparison to control DMSO treated cells. Genes upregulated and downregulated ≥1.5-fold is presented.

Supplementary Table 3: Common significant differentially expressed genes in BAY-850 treated PA-1 and SK-OV3 cells. RNA sequencing data showing all common significant differentially expressed gene in PA-1 and SK-OV3 cell upon treatment with ATAD2 inhibitor BAY-850 5µM for 48 h in comparison to control DMSO treated cells.

Supplementary Table 4: Top 100 common significant differentially expressed genes in BAY-850 treated PA-1 and SK-OV3 cells. RNA sequencing data showing top 100 common significant differentially expressed gene in PA-1 and SK-OV3 cell upon treatment with ATAD2 inhibitor BAY-850 5µM for 48 h in comparison to control DMSO treated cells.

Supplementary Table 5: Significantly downregulated pathways in BAY-850 treated ovarian cancer cells. Function pathway enrichment analysis performed using common significant downregulated gene obtained from RNA sequencing data in PA-1 and SK-OV3 cell upon treatment with ATAD2 inhibitor BAY-850 5µM for 48 h in comparison to control DMSO treated cells.

Supplementary Table 6: Significantly upregulated pathways in BAY-850 treated ovarian cancer cells. Function pathway enrichment analysis performed using common significant upregulated gene obtained from RNA sequencing data in PA-1 and SK-OV3 cell upon treatment with ATAD2 inhibitor BAY-850 5µM for 48 h in comparison to control DMSO treated cells.

Supplementary Table 7: List of Reagents, data and software used in this study with source and identifier.
