# Supplementary material for: ATAD2 is a driver and a therapeutic target in ovarian cancer that functions by upregulating CENPE
Source: Cell Death Dis. 2023 Jul 21;14(7):456. doi: 10.1038/s41419-023-05993-9 (PMC10362061; doi:10.1038/s41419-023-05993-9)
Supplement: Supplementary file 8 — Supplementary Table 7 [file 41419_2023_5993_MOESM8_ESM.pdf]

Supplementray Table 7: Key Resource Table. List of reagents, data, and software used in this study with source and identifier.

**Human primers**		
CDK1	agggcagagtggtggttgtagg	gcgctacctctgcagagtgg
CENPE	gcgggctcaggcaatggaaa	agaggtcaccagcatccgtgt
CENPK	agaagtccggggctggcaag	tggcacccagatcttgaatctcca
CENPN	cgaattgcctggggaacacagt	tggaggaggacgtgaaggcg
CENPU	ggttgtgcggcatggactcc	cgctccgaacagccaatccg
DSN1	actgaggcgggtggattgct	gcctcaacctcctgggctca
PLK4	ccaggcctcggaaggtgtca	ctggcctgggttctccggat
PSMA3	gcccttcctctcgcacctga	cctgcttcttcgccgttccg
PSMC6	accgcttgaggccaggagtt	tcctcatgcctggtcctggc
ZW10	cggcgggtggaggagatcaa	aagatcccggcggacctcac
WEE1	tcggcctttcaagagcccga	ctcctcctcccacgagtcgc
CCNE2	ggtagctggtctggcgaggt	ggattccgtctggctgggct
ACTINB	gtcttcccctccatcgtggg	cctctcttgctctgggcctc
ATAD2	cgcctggagctggatctctc	aaagcacgtgtccggtggta
P53	tatgagccgcctgaggttgg	ggcacaaacacgcacctcaa
P21	gggatgagttgggaggaggc	gaagatcagccggcgtttgg
**shRNAs**	**Clone ID**	
ATAD2 shRNA#1	TRCN0000158771	
ATAD2 shRNA#2	TRCN0000161812	
**Recombinant DNA**		
piggyBac GFP-Luc	Ding et al., 2005	N/A
Act-P-Base	Ding et al., 2005	N/A
**Antibodies**	**Company**	**IDENTIFIER**
PARP	Cell signaling	Cat# 9542S RRID:AB_2160739
Actin	Cell signaling	Cat# 8457 RRID:AB_10950489
ATAD2	Abcam	Cat# Ab244431
CENP-E	Cell signaling	Cat# 96351S RRID:AB_2800260
p53	Cell signaling	Cat#48818 RRID:AB_2713958
p21	Cell signaling	Cat #2947 RRID:AB_823586
CDC2/CDK1	Cell signaling	Cat# #28439 RRID:AB_2798959
CENPK	Invitrogen	Cat# PA5-97579 RRID:AB_2812194
CENPN	Invitrogen	Cat# PA5-100721 RRID:AB_2850225

CENPU	Invitrogen	Cat# PA5-100725 RRID:AB_2850229
DSN1	Invitrogen	Cat# PA5-51742 RRID:AB_2640805
PLK4	Cell signaling	Cat# 71033
PSMA3	Cell signaling	Cat# 2456S RRID:AB_2171417
PSMA6	Invitrogen	Cat# PA5-30134 RRID:AB_2547608
ZW10	Invitrogen	Cat# PA5-62357 RRID:AB_2650417
WEE1	Cell signaling	Cat# 13084T RRID:AB_2713924
CCNE2	Cell signaling	Cat# 4132T RRID:AB_2071197
**Inhibitor**	**Concentrations**	**Source**
BAY850	0.2μM, 1μM, 5μM	selleckchem
GSK923295	10nM,15nM, 25nM	selleckchem
**Commercial assays and kits**		
Click-iT™ Plus EdU Flow Cytometry Assay Kits	Invitrogen	Cat#C10632
FITC-Annexin V Apoptosis Detection Kit	BD Pharmingen	Cat# 556547
CUT&RUN Assay Kit	Cell Signaling Technology	Cat# 86652
BioCoat Growth Factor Reduced Matrigel Invasion Chambers	Corning	Cat #354483
**Deposited data**		
RNA-Seq performed with PA-1 cells treated with BAY-850	This paper	GEO: GSE192673
RNA-Seq performed with SK-OV3 cells treated with BAY-850	This paper	GEO: GSE192673
**Software and Algorithms**		
Prism 9.0	GraphPad	www.graphpad.com/scientific software/prism
ImageJ	https://imagej.nih.gov/ij	N/A
**Cell Lines**		
PA1	ATCC	ATCC CRL-1572
SKOV3	ATCC	ATCC HTB-77
HEK293T	ATCC	ATCC CRL-3216
**Experimental Models: Organisms/Strains**		
Mouse: NSG	Jackson Laboratory	Stock No. 005557
**Chemicals, Peptides, and Recombinant Proteins**		

DMEM	Sigma-Aldrich	Cat# D5796
RPMI	Sigma-Aldrich	Cat# R8758
Fetal Bovine Serum	GIBCO	Cat# 10437-028
Trypsin-EDTA	GIBCO	Cat# 25200-056
Penicillin-Streptomycin	GIBCO	Cat# 15140-122
Effectene Transfection Reagent	QIAGEN	Cat# 301427
VECTASHIELD Hardset Antifade Mounting Medium with DAPI	Vector Laboratories	Cat# H-1500
XenoLight D-Luciferin - K+ Salt Bioluminescent Substrate	Perkin Elmer	Cat# 122799
Agarose, Low gelling	Sigma-Aldrich	Cat# A9045
