# Supplementary material for: ATAD2 is a driver and a therapeutic target in ovarian cancer that functions by upregulating CENPE
Source: Cell Death Dis. 2023 Jul 21;14(7):456. doi: 10.1038/s41419-023-05993-9 (PMC10362061; doi:10.1038/s41419-023-05993-9)
Supplement: Supplementary file 9 — Original Data File [file 41419_2023_5993_MOESM9_ESM.pdf]

## SUPPLEMENTAL MATERIAL

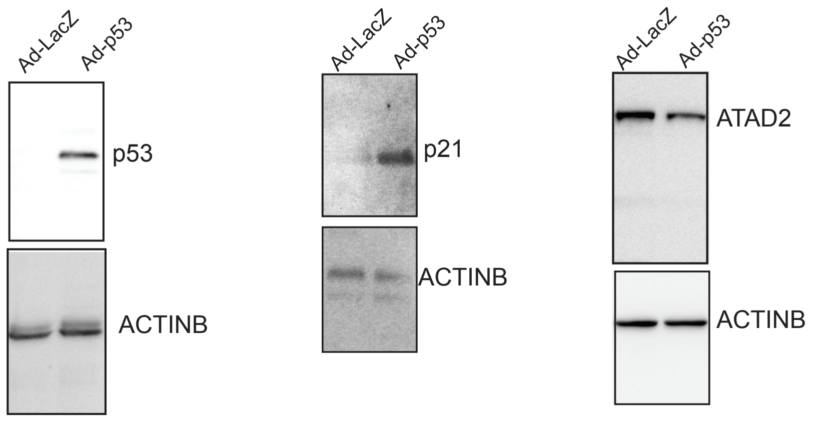


**Raw Immunoblotting uncropped blot images for Fig. 1J**.

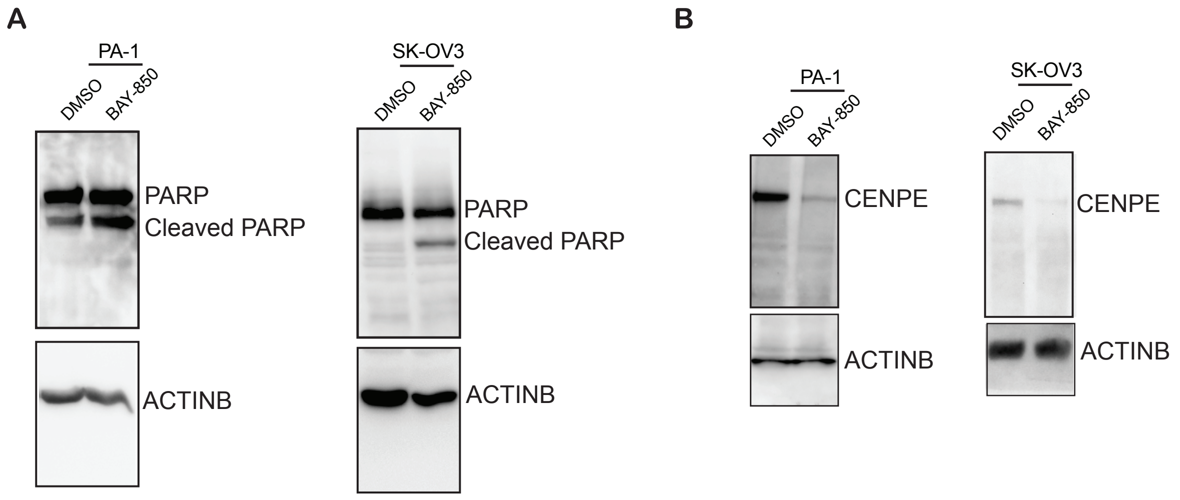


**Raw Immunoblotting uncropped blot images for Fig. 5F and 5I**.
