# Supplementary material for: ATAD2 is a driver and a therapeutic target in ovarian cancer that functions by upregulating CENPE
Source: Cell Death Dis. 2023 Jul 21;14(7):456. doi: 10.1038/s41419-023-05993-9 (PMC10362061; doi:10.1038/s41419-023-05993-9)
Supplement: Supplementary file 10 — Reproducibility checklist [file 41419_2023_5993_MOESM10_ESM.pdf]

SPRINGER NATURE

Corresponding Author Name: Romi Gupta
Manuscript Number: CDDIS-23-104[illegible]

Reporting Summary

Springer Nature wishes to improve the reproducibility of the work that we publish. This checklist is used to ensure good reporting standards and to improve the reproducibility. Please respond completely to all questions relevant to your manuscript. For more information, please read the journal's Guide to Authors.

☑ **Check here to confirm that the following information is available in the Material & Methods section:**

- **The exact sample size (*n*)** for each experimental group/condition, given as a number, not a range
- **A description of the sample collection** allowing the reader to understand whether the samples represent technical or biological replicates (including how many animals, litters, culture, etc.)
- **A statement of how many times the experiment shown was replicated in the laboratory**
- **Definitions of statistical methods and measures**: For small sample sizes (n<5) descriptive statistics are not appropriate, instead plot individual data points
    - Very common tests, such as *t*-test, simple χ^2 tests, Wilcoxon and Mann-Whitney tests, can be unambiguously identified by name only, but more complex techniques should be described in the methods section
    - Are tests one-sided or two-sided?
    - Are there adjustments for multiple comparisons?
    - **Statistical test results**, e.g., ***P* values**
    - Definition of **'center values'** as **median or mean**;
    - Definition of **error bars as s.d. or s.e.m. or c.i.**

Please ensure that the answers to the following questions are reported in the manuscript itself. We encourage you to include a specific subsection in the methods section for statistics, reagents and animal models. Below, provide the page number or section and paragraph number.

Statistics and general methods

	**Reported in section/paragraph or page #**
1. How was the sample size chosen to ensure adequate power to detect a pre-specified effect size? (Give section/paragraph or page #)	All experimenst were conducted in triplicate. See the "MATERIALS AND METHODS" section, subheading "Statistical Analysis" for details.
For animal studies, include a statement about sample size estimate even if no statistical methods were used.	For ATAD2 inhibitor-based experiment: 3 female NSG mice aged 5-6 weeks per experimental group were used for SK-OV3 and PA-1 cell lines.
2. Describe inclusion/exclusion criteria if samples or animals were excluded from the analysis. Were the criteria pre-established? (Give section/paragraph or page #)	Not applicable, because no samples or animals were excluded from the analysis.
3. If a method of randomization was used to determine how samples/animals were allocated to experimental groups and processed, describe it. (Give section/paragraph or page #)	For ATAD2 inhibitor-based experiment: NSG mice were randomly divided into two groups: Vehicle and BAY-850.
For animal studies, include a statement about randomization even if no randomization was used.	3 female NSG mice per experimental group were randomly divided into two group- vehicle or BAY-850

4. If the investigator was blinded to the group allocation during the experiment and/or when assessing the outcome, state the extent of blinding. (Give section/paragraph or page #)	No blinding was done.
For animal studies, include a statement about blinding even if no blinding was done.	The statement is included in the section "MATERIALS AND METHODS" section, subheading “Statistical Analysis" for details.
5. For every figure, are statistical tests justified as appropriate?	Yes. See the "MATERIALS AND METHODS" section, subheading “Statistical Analysis" for details.
Do the data meet the assumptions of the tests (e.g., normal distribution)?	The data meets the assumptions of the tests.
Is there an estimate of variation within each group of data?	See the "MATERIALS AND METHODS" section, subheading “Statistical Analysis" for details.
Is the variance similar between the groups that are being statistically compared? (Give section/paragraph or page #)	See the "MATERIALS AND METHODS" section, subheading “Statistical Analysis" for details.

Reagents	**Reported in section/paragraph or page #**
6. Report the source of antibodies (vendor and catalog number)	Reported in Supplementary Table S7.
7. Identify the source of cell lines and report if they were recently authenticated (e.g., by STR profiling) and tested for mycoplasma contamination	Reported in "Cell culture conditions and reagents" in the Section "MATERIALS AND METHODS".

Animal Models	**Reported in section/paragraph or page #**
8. Report species, strain, sex and age of animals	Reported in "mouse tumorigenesis experiment" in the Section "MATERIALS AND METHODS".
9. For experiments involving live vertebrates, include a statement of compliance with ethical regulations and identify the committee(s) approving the experiments.	All protocols were approved by the UAB Institutional Animal Care and Use Committee. This is reported in the "mouse tumorigenesis experiment" in the section "MATERIALS AND METHODS".

10. We recommend consulting the ARRIVE guidelines (*PLoS Biol.* **8**(6), e1000412,2010) to ensure that other relevant aspects of animal studies are adequately reported.

Human subjects

	Reported in section/paragraph or page #
11. Identify the committee(s) approving the study protocol.	Not applicable
12. Include a statement confirming that informed consent was obtained from all subjects.	Not applicable
13. For publication of patient photos, include a statement confirming that consent to publish was obtained.	Not applicable
14. Report the clinical trial registration number (at ClinicalTrials.gov or equivalent).	Not applicable

15. For phase II and III randomized controlled trials, please refer to the CONSORT statement and submit the CONSORT checklist with your submission.

16. For tumor marker prognostic studies, we recommend that you follow the REMARK reporting guidelines.

Data deposition

	Reported in section/paragraph or page #
17. Provide accession codes for deposited data. Data deposition in a public repository is mandatory for:<br>a. Protein, DNA and RNA sequences<br>b. Macromolecular structures<br>c. Crystallographic data for small molecules<br>d. Microarray data	Reported in "Supplementary Material-Supplementary Table 7":<br>RNA-seq data was submitted to Gene Expression Omnibus (GEO): GSE192673.

Deposition is strongly recommended for many other datasets for which structured public repositories exist; more details on our data policy are available in the Guide to Authors. We encourage the provision of other source data in supplementary information or in unstructured repositories such as Figshare and Dryad. We encourage publication of Data Descriptors (see Scientific Data) to maximize data reuse.

18. If computer code was used to generate results that are central to the paper's conclusions, include a statement in the Methods section under "**Code availability**" to indicate whether and how the code can be accessed. Include version information as necessary and any restrictions on availability.	Not applicable
